# Can chatbots teach us how to behave? Examining assumptions about user interactions with AI assistants and their social implications

**DOI:** 10.3389/frai.2025.1545607

**Published:** 2025-05-14

**Authors:** Eleonora Lima, Tiffany Morisseau

**Affiliations:** ^1^Trinity College, Centre for Digital Humanities, Dublin, Ireland; ^2^Université Paris Cité and Univ Gustave Eiffel, LaPEA, Boulogne-Billancourt, France; ^3^Strane Innovation, Gif-sur-Yvette, France

**Keywords:** AI assistants, gender inclusivity, human-AI interaction, intentionality, harassment and abuse, gender bias

## Abstract

In this article we examine the issue of AI assistants, and the way they respond to insults and sexually explicit requests. Public concern over these responses, particularly because AI assistants are usually female-voiced, prompted tech companies to make them more assertive. Researchers have explored whether these female-voiced AI assistants could encourage abusive behavior and reinforce societal sexism. However, the extent and nature of the problem are unclear due to a lack of data on user interactions. By combining psychological and socio-cultural perspectives, we problematize these assumptions and outline a number of research questions for leveraging AI assistants to promote gender inclusivity more effectively.

## Introduction

In 2017, at a time when the MeToo Movement was reaching global attention, some journalists (Buxton, 2017; Fessler, 2017a; Fiegerman, 2017; Mitchell, 2017) discovered that, when addressed with insults and sexually explicit requests, AI assistants such as Alexa, Siri, Cortana, and Google Assistant tended to respond in a submissive and coy way. This was understandably presented as yet another proof of the sexist society that we live in. The public outrage that ensued against what was perceived as a sexist stereotype—as most of these AI assistants had female voices and characterizations—pressured tech companies to take measurements and rectify the situation.^1^

In the following years, a vast number of scholarly publications discussed the issue: whether and why AI assistants' female characterization encouraged insults and abusive talk, whether this trend mirrored existing sexist behaviors in society and even reinforced them, and what could be done to solve the problem (e.g. Phan, 2019; Schiller and McMahon, 2019; Walker, 2020; Jiang, 2021; Oliveira and Amaral, 2021; Strengers and Kennedy, 2021; Rhee, 2023; Borau, 2025). In 2019, even UNESCO published a document entitled *I'd blush if I could: closing gender divides in digital skills through education*, the title of which refers to one of Siri's infamous responses to the insulting statement “Hey Siri, you are a bitch” (West et al., 2019).^2^ The bottom line, paraphrasing the title of one of these academic studies, was that *Siri, Alexa, and other smart home devices need a feminist reboot* (Strengers and Kennedy, 2021). Companies responded to this public outrage by changing the ways in which their AI assistants answered, making them more assertive or even scolding abusive users and teaching them about the impact of verbal harassment, especially on women.

While the concerns are legitimate and the spirit of initiatives designed to counteract gender discrimination and verbal harassment laudable, the discourse on insults to AI assistants relies on a number of assumptions about the scale and nature of the issue, which needs further investigation. For one thing, conversations between individuals and their AI assistants are not publicly available, making it impossible to determine the extent of the problem. The nature of the insults also remains unclear without a contextual background. Such “insults” could be playful or could be an innocent way for people to release tension, like other forms of cursing and swearing (Husain et al., 2023). In addition, the negative assessment of these behaviors toward AI presupposes a correspondence between individuals' interactions with an AI agent and their interactions with other people. Either because people who insult their female-voiced assistant are assumed to do the same when talking to women (Moradbakhti et al., 2022), or worse, because it is believed that this behavior reinforces individuals' latent sexism and makes them desensitized to the effects of verbal harassment (Keijsers et al., 2021). However, there is low evidence that people in real life will adopt the toxic behavior they exhibit when interacting with AI assistants. A parallel can be drawn with the issue of video games, whose negative effects on people's personalities and violent tendencies have now been largely reassessed (Dill and Dill, 1998; Ferguson, 2007; Ferguson et al., 2015).

This lack of clarity about the scale and nature of the problem calls into question the strategies adopted to address it. If insults to AI assistants are not as rampant as they appear to be, pressuring tech companies to fix the problem by designing more assertive—and potentially patronizing—chatbots could provoke negative reactions rather than increase user awareness. Not to mention that it would also mean investing a lot of money and energy on a nonexistent problem, resources that could be spent on other more pressing issues, such as sexism and gender exclusion in the tech world.

The purpose of this article is to discuss the above limitations and to outline the research questions that remain to be answered to address the issue, including: what kind of response should be expected when someone is confronted by a chatbot? Is there really a need for more assertive and empowered AI assistants? What social role and purpose could AI assistants play in promoting gender equality?^3^ Far from downplaying the role of technology in enforcing and reinforcing hierarchies of power and exploitation, our goal is to question the efficacy of the current approach aimed at making AI assistants “feminist” by asking tech companies to programme them to be more assertive if not combative. In an interview on the topic of feminism as a design methodology in HCI, Alexa Ahmed and Lilly Iranu quote activist and philosopher Angela Davis who states: “Feminism involves so much more than gender equality. And it involves so much more than gender. Feminism must involve a consciousness of capitalism” (Ahmed and Irani, 2020). In attributing too much importance to the tone, voice, and persona of AI assistants and in giving tech companies the power to easily fix sexism by reprogramming them without questioning the entire design, we risk ignoring the economic and power structure underlying the problem, and offering tech companies an easy way to rebrand themselves without truly engaging with feminist HCI principles (Bellini et al., 2022).

In our analysis we adopt a pluridisciplinary perspective, relying on psychological concepts as well as on insights from the Science and Technologies, to capture both the cognitive and socio-cultural aspects of the question. In what follows, we offer a theoretical analysis of how insults to AI assistants should be dealt with, as well as a set of recommendations on how best to harness the power of AI assistants to promote gender inclusivity.

## Are insults to AI actually a problem?

Discussions about users' insults to AI assistants stem from the assumption that people's interaction with these chatbots mirrors their exchanges with other human beings. This belief is reinforced by the fact that most AI assistants are designed to be perceived as female: they generally have a default woman voice or a female-sounding name—like in the case of Siri, Alexa and Cortana—and are therefore addressed by users as if they were female entities. Verbal harassment against women is rampant online—from social media to online multiplayer games—and it is fuelled by anonymity. Insults against a female-sounding AI assistant are thus understood to fall into the same category as cyberbullying. While we obviously recognize the gravity of the latter, we believe that it is important to challenge this equivalence by understanding the real scale, motivations, and impact of “insulting” an AI assistant.

###  There is no direct evidence of people's insulting AI assistants

First of all, while it is relatively easy to assess the scale of online harassment against women, as insulting messages are available on any social media platform and forum, the prevalence of insults directed toward AI assistants cannot really be measured. For obvious reasons, companies providing these services—such as Apple, Amazon, Google—do not release the conversations that their customers entertain with their chatbots. Rather, the studies that have been published on this subject are based on interactions that were designed for research purposes. For example, researchers will write a list of offensive or sexually oriented questions and comments, usually based on language and interactions seen online, and test the responses of various AI assistants (Fessler, 2017b; Cercas Curry and Rieser, 2018; Oliveira and Amaral, 2021; Jiang, 2021). In other cases, subjects are asked to evaluate these exchanges—the investigator's questions and the chatbot's answers—and express their level of amusement, discomfort, or disapproval (Lopatovska, 2020; Pinelli et al., 2023). Undoubtedly, these studies provide important insights into the design of AI assistants and explain how anthropomorphism and genderization shape users' perceptions. But they do not shed light on the true extent and nature of user insults to AI assistants.

Addressing this lack of direct empirical evidence requires technology companies to actively collaborate with researchers to analyse interaction data in a systematic way. Quantitative studies could then precisely establish the prevalence and frequency of insults toward AI assistants in authentic user contexts. Such studies would shed light on whether users genuinely attribute human-like agency and intentions to virtual agents, or regard them as convenient targets for harmless, inconsequential interactions.

###  AI-directed insults are not necessarily what we think they are

The lack of user data also means a lack of contextual background, which leads to a second problem, namely the difficulty in assessing the true nature of these interactions. Veletsianos et al. (2008) describe how people may display playful intentions when they insult and provoke AI agents. Brahnam and De Angeli (2008) write that “[i]ndependent of the context, people appear to enjoy provoking, teasing, and often humiliating the conversational partners. The studies also show an example of a creative misuse of technology. Some users appropriate the conversational agent, transforming it into a toy for practicing proposing sex. This toy seems to be conducive toward disinhibited behavior, as it does escape traditional moral constraint.” While it would be misguided to assume that every insult to an AI assistant has a humorous and playful intention, it is certainly an aspect that needs to be taken into consideration when trying to understand this issue. A quick search on Amazon's websites in various European countries shows how several free “Alexa skills” created and published by developers, often amateurs, are devoted to offensive humor. Most of these skills allow Alexa to insult, tease, roast or even respond to users' requests in an insolent—and juvenile—manner, such as belching or passing gas.

While in these cases it is the AI assistant that is insulting its users rather than the other way around, this is indicative of the playful attitude described by Brahnam and De Angeli, and the pleasure that people might find in teasing and being teased. Considering that insults to AI assistants sometimes fall within the realm of humor [a “positive and adaptive response to benign offense,” as McGraw and Warren (2010) describe it] puts things in a very different light. These behaviors could in fact be part of a coping mechanism used by people to feel more comfortable when confronted with artificial agents.^4^

Given the ambiguity regarding the meaning and motivation of insults to AI assistants, qualitative approaches (e.g. user interviews, observational studies and ethnographic methods) would be highly valuable. Such approaches would make it possible to clarify the complex interactions between individuals' perceptions of conversational agents, their intentions (playful, exploratory, aggressive, humorous), and the social contexts that influence these interactions. This is a crucial step toward distinguishing between genuinely problematic behavior and behavior associated with more benign, adaptive forms of technological engagement.

###  The impact of people's behavior toward AI assistants on their real-life interactions is unclear

Even if such behavior were to be widespread, would they be a problem in the first place? They would be if someone else was hurt by the insult (which is obviously not the case for the target AI assistant, although we discuss below the circumstances in which sexism toward AI could foster a sexist culture), or if these AI-directed interactions lead users to display the same behaviors toward real persons online—which is what happens in the case of harassment of customer service staff and cyberbullying, to which this problem is often compared (Chin et al., 2020). So is it the case that people do not really distinguish between human and artificial agents?

The Computers Are Social Actors (CASA) paradigm answers this question in the affirmative. It argues that people's interactions with computers are fundamentally social, reflecting the way they interact with other people (Reeves and Nass, 1996). According to this paradigm, our interaction with computer technologies is fundamentally different from the one we have with a car or a toaster, because of their ability to mimic human intelligence. From this point of view, swearing at an AI assistant is not the same as swearing at our car when it breaks down on the way to work. To investigate this question, De Angelis and Braham conducted an empirical study of people's conversations with the Jabberwacky chatterbot, and provide an in-depth look at the particular interactions between humans and AI (De Angeli and Brahnam, 2008). They observed that insults and sexual comments directed at the chatbot were different from the curses that people usually reserve for unresponsive or inefficient tools. Yet, such behaviors are also way more frequent and aggressive than in any human-to-human conversation. De Angelis and Braham concluded that “users were aware their words were harmless.” They suggested that “people treat talking computers less as they do people and more as they might treat something not quite an object and yet not quite human.”

It is worth mentioning, however, that since the publication of this study, HCI and interhuman communication have changed a lot. Today, exchanges between people are often developed through interfaces that closely resemble those used to interact with AI assistants and other automated services, something that may dilute the contextual clarity of any of the interactions.^5^ In a more recent study, Strait et al. (2018) found that users who were abusive toward robots were also more frequently abusive in their general tweeting, but concluded that disinhibition toward robots reflected individual differences in antisocial tendencies rather than a generalized phenomenon. What these interactions truly say about individuals' behavior with other people remains unclear.^6^

Critically assessing the transferability of aggressive interactions in AI contexts to real-world interactions is a fundamental line of research. Clarifying in what context and why individuals apply the same heuristics in their interactions with humans and with AIs will not only fuel important theoretical debates in psychology, but will also have important ethical implications for how AIs should be programmed to respond to human demands. To address these questions empirically, controlled experiments comparing user interactions with humans vs. artificial agents should be combined with longitudinal studies to assess whether aggressive behavior directed at AI assistants is subsequently manifested in interactions with real people.

## In what ways could sexist behavior toward AI be harmful?

Exposure to a sexist context can significantly impact the acceptance of such attitudes. For instance, empirical evidence suggests that the presence of sexist content in digital games influences players' attitudes and behaviors, reinforcing gender biases and perpetuating discriminatory norms (Tompkins and Lynch, 2018). Numerous studies in social psychology (both observational and experimental) show that exposure to sexist environments has two main effects:

1) *It influences the moral evaluation of these attitudes*. For example, Ford et al. (2013) found that sexist humor creates a context that justifies the expression of prejudice against women. Their study investigated the social consequences related to sexism and revealed that men with higher levels of hostile sexist attitudes were more likely to express beliefs justifying the gender status quo after exposure to sexist humor, compared to exposure to neutral humor or non-humorous sexist material. Douglas and Sutton (2014) also observed a strong gender difference in attitudes toward sexist language that was significantly mediated by gender-specific system justification and social dominance orientation.
2) *It increases the propensity for antisocial behavior and social disengagement* (Bandura, 1991, 2002), by making arguments justifying such behavior more salient. These environments thus amplify discriminatory attitudes and behaviors, leading to a higher likelihood of social and moral disengagement among individuals exposed to such contexts (Bohner et al., 2005; Paciello et al., 2021).

In the case of a user interacting with a female-voiced AI assistant, though, the situation is different. In this situation, the user is interacting with a non-human agent, a context that is not a priori conducive to reinforcing a social norm. When the interaction is not private, however (that is, the agent is not alone), sexist references and attitudes will presumably be interpreted as referring to interactions with real women, and perceived either as normal behavior or as a joke intended for a social group. If there is no ambiguity about the social norms shared between the individuals present, and if these norms are not sexist, then insults directed at an female-voiced AI assistant would have little negative impact in terms of spreading toxic attitudes. But that can hardly be the case. Such cognitive opacity can encourage the perpetuation of social norms that run counter to respect for others.

Another reason why these insults could be a problem is that an AI assistant's reactions say something about the people who programmed it. AI is a socio-technical system, and it is an expression of what is or is not acceptable in our society. Cases where female AI, such as Siri, are the target of gendered verbal abuse may actually be contributing to the perpetuation of sexist behavior through the way the AI assistant reacts to it. AI responses can crystallize patterns of toxic relationships because they provide a sense of normality induced by responses that are taken for granted—even though it is only a choice made by the developer.

## What are the risks of regulating violent and sexist behavior toward AI?

There is certainly a strong need to mobilize research aimed at improving social relations and reducing the prevalence of sexist behavior. The toxicity observed in interactions on platforms such as X illustrates the extent to which virtual environments can foster antisocial behavior. Although addressing the social norms that are prevalent in many online communities should be part of this effort, regulating people's behavior toward AI would probably worsen the problem.

After the tendency of AI assistants to respond to sexual and abusive comments in a coy, flirtatious, or submissive way was brought to public attention in 2017, a series of actions were taken by tech companies to correct this and conform to users' sensitivity. For instance, Amazon added a specification in Alexa guidelines about the assistant not having a gender (Abercrombie et al., 2021). In 2020, Accenture Lab created the first synthetic non-binary voice assistant.^7^ And in 2021, Apple eliminated Siri's default female voice (Panzarino, 2021). However, it was felt that simply avoiding the portrayal of AI assistants as female was not enough to curb sexism. In response to the already mentioned UNESCO report *I'd blush if I could*, a number of initiatives supported the idea of having unapologetically assertive AI assistants calling out their abusive users. This was the case, for example, with the Shut Up Sexism campaign launched by Unilever's Lux beauty brand in 2022, which created a skill for Amazon's Alexa and Google Assistant that prompted the voice assistants to respond to insults with insults (Schwarz, 2022). More recently, in March 2024, Amazon Italia partnered with NGO ActionAid to develop a skill that allows Alexa to respond to insulting and abusive comments or questions by explaining how this behavior qualifies as sexism and offering data on the impact and dangers of verbal abuse on women.^8^ The skill was released on International Women's Day and received a lot of media attention.

While these initiatives clearly have the best of intentions, they are likely to be counterproductive.

First, rather than learning from these interactions, people may reject the message altogether, perceiving it as a form of control over their behavior. They may respond with a form of psychological reactance, motivated by a sense of constraint and aimed at restoring some degree of freedom (Brehm and Brehm, 2013). This reactive behavior could therefore create a backlash, i.e. motivate users to adopt the attitude that was intended to be discouraged. A recent empirical study involving 1,486 Chinese students found negative reactions to overtly positive gender representations, which were perceived as patronizing and prejudicial by both male and female participants in the experiment (Wang et al., 2024). Programming an AI assistant to overtly call out offensive or sexist comments, or to overtly support feminist views, poses similar risks.

Second, initiatives such as Amazon Italy's and ActionAid's rely on an equivalence between a female-sounding chatbot and women's experiences of abuse, an implicit assumption in any claim denouncing sexist behavior toward AI assistants: insulting Alexa is not too dissimilar to harassing a woman online or, at least, it should be punished as such. The genderization of AI agents, combined with campaigns that further exploit the comparison between women and robots can lead to what Erscoi et al. (2023) call “diminishment via false equivalence.” This is what happened, for instance, when in 2019 CGI Instagram influencer Miquela shared a video about a case of sexual harassment “she” experienced (Song, 2019), or when in 2020 other Black-presenting CGI influencers offered their views on racism during the Black Lives Matter protests period (Sobande, 2021): in both cases, people were deeply disturbed, and criticized the initiative as exploitative of people's real-life sufferings.

Finally, these initiatives and changes to the way AI assistants sound and respond to insults can be perceived as pink-washing strategies adopted by companies to retain customers and avoid public scrutiny. Tech companies' efforts to combat sexism may be challenged by the fact that 72% of women in tech roles have experienced at least one form of sexism at work, according to a 2023 survey by The Fawcett Society (Ville, 2023). Focusing heavily on users insulting AI assistants may contribute to overshadowing more pressing and serious forms of gender discrimination.

## Can AI play a beneficial role in limiting offensive behavior on the Internet?

There is a long way to go in order to make AI more gender inclusive, and to fight potential biases that would perpetuate through the way AI-based systems are made (Simon et al., 2020). A number of proposals have been made to tackle the problem but focusing on how men interact with AI is not one of them. Yet, AI assistants systems could be improved to promote more inclusive and respectful behaviors online.

###  Female AI assistant should not contribute to perpetuate anti-feminist priors

The risk is not that users will take the behavior of an AI assistant as another typically female response that will justify the persistence of stereotypes. But stereotypical answers say something about the shared representations of women, and the normalization of these stereotypes should be challenged. Ensuring that female voices in AI do not conform to sexist norms would represent a significant advancement. While existing sexist biases in AI systems can negatively impact women, they also have the potential to promote gender equality. For instance, feminist social robot behaviors have been found to enhance girls' perceptions of robot credibility while reducing gender bias in boys (Winkle et al., 2021). This leaves open the question of how AI should respond to interactions, and there is probably more than one way to go.

###  AI assistants should not be perceived as being the tool of designers with a political agenda

Any attempt to educate people in spite of themselves carries a high risk of psychological reactance. In that respect, the neutrality of AI is an asset. By definition, an AI has no personal opinion, and may therefore be considered as “neutral.” For this to be the case, no intention of winning a case should be perceived: when no anti-social intention is perceived, the risk of negative emotions is decreased, and anti-social reactions are less likely to happen. AI could thus help to pacify debates, by virtue of not having to express a personal opinion: it could lead people to be more critical of their own arguments, as social or emotional stakes would be reduced. Chatbots could thus be designed to disseminate accurate information and engage in polite, constructive dialogue. For example, they would not react by over-interpreting the user's intentions and, their responses would not be perceived as defensive. The issue of domination should not be addressed in advance, as such efforts reinforce implicit boundaries. Examples of “neutral” responses to potentially offensive content might be: “This could be misinterpreted”/“I have heard some people refer to women in such a way, which in many contexts can be perceived as aggressive.”

###  Promotion of gender equality should be grounded on explicit assumptions

If what an AI assistant says departs from what is generally accepted as neutral, the developers' intention should be explicitly acknowledged as such. If the intention behind a chatbot is to promote better behavior, this intention should be transparent (or it could be presented as an experimentation). Nudging people into behaving better is fair as long as there is no ambiguity that the goals of both the target person and the nudge designer are aligned and recognized as such. Otherwise, boosting (that is, working on people's ability to make their own choices) should be preferred (Hertwig and Grüne-Yanoff, 2017). In the case of sexist behavior, there is no debate that respectful behavior should be encouraged. The debate is whether AI assistants should be a tool to teach people how to behave. Alternatively, AI could more simply be used as a tool in training environments and presented as such.

###  AI's ability to recognize other people's intentions and adapt its speech could be harnessed

Finally, AI will soon be able to pick up on the tone of speech, as research into speech emotion recognition advances and such systems are implemented in commonly used AI tools—albeit with variable degrees of success (see Dhekale et al., 2023; Anthony and Patil, 2023). If this is indeed the case, algorithms could respond proactively, before the user's defensiveness becomes too dominant, by anticipating tense situations and responding in a way that restores a reassuring climate.

## Conclusion

Fearing that online sexism will escalate offline is justified. Yet, focusing on how men interact with AI is probably not an efficient strategy. This article aimed to clarify the boundaries between legitimate fears and speculations, as well as the conditions for the beneficial influence of AI-based activism. To confirm the value of AI-driven initiatives in addressing these issues, more research is needed, answering the questions raised in this article: first, a systematic assessment of the prevalence of insults to AI Assistants; second, a thorough analysis of the motivations underlying these interactions; and third, a rigorous evaluation of potential repercussions on real-life interactions.

## Data Availability

The original contributions presented in the study are included in the article/supplementary material, further inquiries can be directed to the corresponding author.

## Footnotes

^1^The 2018 online petition “Siri and Alexa Should Help Shut Down Sexual Harassment” signed by over 150,000 people is an example of such reaction. This petition suggested to “take a new approach entirely, and say something like ‘That's not an okay thing to say to me.' Even better—what if Siri and Alexa met sexual harassment with actual stats about its prevalence?” Available here: https://www.thepetitionsite.com/246/134/290/ (accessed: 6 December 2024).

^2^The UNESCO report opens with this statement: “The title of this publication borrows its name from the response given by Siri, a female-gendered voice assistant used by hundreds of millions of people, when a human user would tell ‘her', ‘Hey Siri, you're a bi***.' Although the AI software that powers Siri has recently been updated to reply to the insult more flatly (‘I don't know how to respond to that'), the assistant's submissiveness in the face of gender abuse remains unchanged since the technology's wide release in 2011.”

^3^There are two reasons why we focus on AI assistants rather than conversational systems more generally, which have grown in popularity in recent years. First, our analysis is a response to the nearly decade-long debate about AI assistants and the sexist assumptions that guide their design. Second, what makes AI voice assistants different from AI conversational systems is their function. Their purpose is to serve and assist users, and their caring and accommodating tone and persona is the reason they are—or at least were—characterized as female by default, reinforcing the stereotype of the female secretary to the male boss.

^4^McGraw and Warren also note that: “Humor provides a healthy and socially beneficial way to react to hypothetical threats, remote concerns, minor setbacks, social faux pas, cultural misunderstandings, and other benign violations people encounter on a regular basis” (McGraw and Warren, 2010).

^5^We thank the anonymous reviewer for this valuable insight.

^6^Particularly instructive in this context is the literature on a similar topic, the example of video games. While numerous studies have linked violent video games to increased aggressive behavior and cognitions (Lee et al., 2010; Kristiawan et al., 2023), the relationship between violent video games and real-life behavior is complex and not fully understood, with other factors serving as far more powerful predictors of violent behavior than video games (Gunter and Gunter, 2016; Markey et al., 2015). On the other hand, sexist behaviors toward AI assistants, as in the case of players' attitudes in the context of video games, may in some cases, contribute to the perpetuation of sexist automatisms.

^7^Here is the video illustrating the project: https://www.youtube.com/watch?v=mL1n5AEFLl4&t=1s.

^8^Here is the page illustrating the initiative on Amazon.it: https://www.aboutamazon.it/notizie/dispositivi-amazon/alexa-al-fianco-di-actionaid-contro-la-violenza-verbale.
